# LACK OF SLEEP AND PERCEIVED SOCIAL ISOLATION

**DOI:** 10.1093/geroni/igae098.2256

**Published:** 2024-12-31

**Authors:** Emma Vass, Amy Fiske

**Affiliations:** West Virginia University, Morgantown, West Virginia, United States; West Virginia University, Morgantown, West Virginia, United States

## Abstract

As F. Scott Fitzgerald wrote, “The worst thing in the world is to try to sleep and not to.” Insomnia is related to multiple distressing health outcomes. Research suggests that one of these outcomes may be higher levels of loneliness (Hom et al. 2017). The aim of this study was to use secondary data analysis to examine the relationship between sleep duration and perceived social isolation among older and younger adults. This study hypothesized that shorter sleep duration would lead to greater perceived social isolation. A sample of 147,694 participants ages 18-64 and 87,343 participants ages 65-99, from the 2022 Behavioral Risk Factor Surveillance Survey (BRFSS) was used to conduct a linear regression analysis on the relationship between these two variables after controlling for gender and education level. The overall model was significant for both older and younger adults: older, R² =.027, F(3, 87339) = 433.41, p <.001; younger, R² =.015 F(3,147690) = 1343.32, p <.001. Regression coefficients were significant for both older, β=.099, p<.001, and younger, β=.142, p<.001, adults. Study results indicated that lower sleep duration is related to higher perceived social isolation in both older and younger adults. Nonetheless, effect sizes were small. With this information in addition to the aforementioned previous work, we can continue to explore the connection between loneliness and sleep problems.

